# Retrospective analysis of adjuvant treatment for localized, operable uterine leiomyosarcoma

**DOI:** 10.1002/cam4.4665

**Published:** 2022-03-20

**Authors:** Jomjit Chantharasamee, Karlton Wong, Pasathorn Potivongsajarn, Amir Qorbani, Neda Motamed, Sandra Brackert, Joshua Cohen, Bartosz Chmielowski, Anusha Kalbasi, Jianyu Rao, Scott Nelson, Arun Singh

**Affiliations:** ^1^ Division of Hematology‐Oncology, Department of Medicine University of California Los Angeles California USA; ^2^ Division of Medical Oncology, Department of Medicine, Siriraj Hospital Mahidol University Bangkok Thailand; ^3^ Department of Pathology University of California Los Angeles California USA; ^4^ Division of Gynecologic Oncology, Department of Obstetrics and Gynecology University of California Los Angeles California USA; ^5^ Department of Radiation Oncology University of California Los Angeles California USA

**Keywords:** leiomyosarcoma, operable, uterine leiomyosarcoma, uterine sarcoma

## Abstract

**Objective:**

Currently, there is no standard adjuvant treatment protocol for localized uterine leiomyosarcoma (uLMS) as clinical trials to address this question have been retrospective, underpowered, or undermined by slow accrual rates. The aim of this study is to determine the benefit of adjuvant chemotherapy for uLMS.

**Methods:**

We reviewed the medical records of localized uLMS patients who had underwent adjuvant therapy after upfront surgery between 2000 and 2020. The cases were blinded for review. We evaluated the influence of various clinical characteristics and different types of adjuvant therapies on specific outcomes.

**Results:**

Sixty‐eight patients (median age: 50 years) were included for analysis. Forty of 68 (58.8%) patients received adjuvant chemotherapy +/− radiation therapy and 25 patients (38.6%) did not receive any adjuvant therapy. At a median follow‐up time of 43.3 months, 45 patients (66.1%) had relapsed disease. The median disease‐free survival (mDFS) for all patients was 23.1 months. Patients who received any adjuvant treatment (chemotherapy and/or radiation) trended toward a longer mDFS compared with those who did not receive any adjuvant therapy (29.7 vs. 14.1 months, *p* = 0.26). Patients who received adjuvant chemotherapy alone had a longer, but nonstatistically significant mDFS compared with those who did not receive any adjuvant treatment (22.2 vs. 14.1 months, *p* = 0.18). Additionally, univariate analysis found that tumor size large than 10 cm, and a mitotic rate >10/10hpf were independent prognostic factors for worse DFS.

**Conclusions:**

Though DFS was more favorable among those who received adjuvant therapy, it was not statistically significant, and thus based on this data adjuvant therapy for resected uLMS is still in question.

## Abstract 2

Determined the benefit of adjuvant therapy for operable uterine leiomyosarcoma. No survival benefit of adjuvant chemotherapy.

## 1 INTRODUCTION

Uterine leiomyosarcoma is the most common sarcoma of the uterus; however, it is quite rare, representing only 3%–9% off all malignant uterine tumors.^1^, ^2^ While the majority of uLMS patients are diagnosed at an early disease stage and undergo complete resection, the 5‐year recurrence rate remain high, ranging from 45% to 82%.^3^, ^4^, ^5^, ^6^, ^7^ Previous studies showed that independent prognostic factors such as high mitotic rate, large tumor size, and advanced age were associated with shorter survival.^8^, ^9^, ^10^ However, no prognostic factor have been established to identify patients who might benefit from adjuvant therapy.^3^, ^8^ Several phase II single‐arm studies demonstrated low rates of recurrence when adjuvant chemotherapy is administered.^11^, ^12^, ^13^ Hensley et al. reported that 78% of patients remain disease‐free at 2 years in operable stage I‐III uterine leiomyosarcoma receiving four cycles of gemcitabine and docetaxel.^11^ However, these studies did not employ a control arm, and relied on historical data to demonstrate relative survival benefit with adjuvant chemotherapy. Randomized studies of adjuvant chemotherapy/radiation in patients with uLMS were insufficiently powered to show survival advantage.^5^, ^14^, ^15^, ^16^ A multicentric, randomized‐controlled trial reported that the estimated overall survival of observation tend to be longer than adjuvant chemotherapy arm, however, this study was closed prematurely due to accrual futility and thus was not able to make any definitive conclusions regarding adjuvant chemotherapy.^16^ Giuntoli and Rosanna reported retrospective studies of patients with resectable uLMS, and no benefit of adjuvant chemotherapy on survival outcome was discovered.^8^, ^17^ On the other hand, Tzu et al. demonstrated the overall survival benefit of adjuvant chemotherapy in a retrospective study of 51 patients.^3^ Similar to a retrospective study by Ricci et al., receiving adjuvant chemotherapy prolonged overall survival in multivariate analysis, however, no data of baseline prognostic factors by adjuvant chemotherapy status were reported.^18^

To this day, the role of adjuvant therapy after complete resection remains controversial. Herein, we report the clinical characteristics and outcomes of patients with operable uLMS treated in a single sarcoma referral center to investigate the impact of adjuvant treatment on survival outcome and prognostic factors that may influence survival.

## 2 MATERIALS AND METHODS

### 2.1 Patient selection

This retrospective analysis was conducted at a single institution. We reviewed the medical records of all patients with operable, FIGO stage I‐III uLMS who underwent upfront surgery between 2000 and 2020. The cases were blinded for review. We evaluated the influence of demographic data, type of surgery, tumor size, mitotic index, margin status, stage, and adjuvant therapy on survival outcomes. Patients who: (i) were inadequately staged, (ii) received neoadjuvant treatment, and (iii) had metastatic disease on presentation were excluded.

### 2.2 Statistical analysis

Patient characteristics and treatment outcomes were analyzed using descriptive statistics. Kaplan–Meier survival analysis was used to estimate DFS and inter‐group comparisons were analyzed by log‐rank test. DFS was defined as the interval between the date of definitive surgery to the date of disease recurrence or last follow‐up or death, whichever occurred first. Variables that were independently associated with DFS in univariate analysis (with a *p*‐value less than 0.05) were included in multivariate analyses by Cox proportional hazard regression using enter regression method. A two‐tailed *p*‐value <0.05 was considered statistically significant for all tests. All analyses were carried out using SPSS version 18.

## 3 RESULTS

Sixty‐eight patients (median age: 50 years) were analyzed; the median time from surgery to adjuvant treatment was 44 days. Sixty‐four of 68 (94.1%) patients underwent total abdominal hysterectomy with bilateral salpingo‐oophorectomy (TAH‐BSO). A total of 63.2% were FIGO stage I, 17.7% were stage II and 8.8% were stage III. Morcellated or fragmented tumors were found in 12 (17.6%) patients. The median tumor size was 11 cm (range: 3–24.5 cm) and the median mitotic rate was 14 mitoses/10 high‐power fields (HPF), (range: 1–63). Forty of 68 (58.8%) patients received adjuvant chemotherapy +/− radiation therapy. Thirty‐three of 40 (82.5%) patients received a combination of gemcitabine at starting dose of 900 mg/m^2^ on D1, D8, and docetaxel at starting dose of 75 mg/m^2^ for 6 cycles, and four of 40 (10%) received four cycles of gemcitabine and docetaxel followed by four cycles of doxorubicin at starting dose of 75 mg/m^2^. In total 62.5% of patients completed planned chemotherapy. The median number of chemotherapy cycles was 6.25 (38.6%) patients did not receive any adjuvant treatment. Two patients underwent adjuvant radiation alone, and one patient received adjuvant letrozole alone (Table 1). In total there were 18 patients with FIGO stage II or III disease; 15/18 (83.3%) received adjuvant chemotherapy. (Table 3)

**TABLE 1:** Patient and disease characteristics of localized uterine leiomyosarcoma

Characteristics	*N* = 68
Age median (Range) (years)	50 (25–76)
Type of surgery, *n* (%)	
TAH with BSO	55 (80.9%)
Total hysterectomy	3 (4.4%)
Supracervical hysterectomy	1 (1.5%)
TAH with BSO and extended debulking surgery	9 (13.2%)
Morcellated specimen, *n* (%)	11 (16.1%)
Median tumor size (range), cm	11(3–24.5%)
Missing data, *n* (%)	11 (16.1%)
Median mitotic rate/10HPF (range)	14 (1–63)
Missing data, *n* (%)	16 (23%)
Margin, *n* (%)	
R0	38 (55.9%)
R1	7 (10.3%)
R2	4 (5.9%)
Not assessable margin	19 (27.9%)
Fragmented specimens or morcellation	12
Undocumented margin status	7
FIGO stage, *n* (%)	
I	43 (63.2%)
II	12 (17.6%)
III	6 (8.8%)
Missing data	7 (10.3%)
Any adjuvant treatment, *n* (%)	43 (63.2%)
Adjuvant radiation alone, *n*	2
Adjuvant letrozole alone, *n*	1
Adjuvant chemotherapy, *n* (%)	40 (58.8%)
Chemotherapy alone	30 (44.1%)
Chemotherapy plus radiation	10 (14.4%)
Chemotherapy agent, *n* (%)	
Gemcitabine + Docetaxel	33 (82.5%)
Gemcitabine + Docetaxel followed by Doxorubicin	4 (10%)
Chemoradiation with Ifosfamide followed by Gemcitabine + Docetaxel	1 (2.5%)
Aldoxorubicin	1 (2.5%)
Immunotherapy (unknown)^a^	1 (2.5%)

Abbreviations: BSO, bilateral salpingo‐oophorectomy; TAH, total abdominal hysterectomy.

a Treatment outside U.S.

At a median follow‐up of 43.3 months, 45 (66.1%) patients had disease relapse. The most common sites of recurrence were the pelvic cavity, which was seen in 18 (40%) patients followed by eight (17.7%) patients with lung‐only metastases. The mDFS for the entire cohort was 23.1 months. The median time to recurrence was 24.9 months, 36.6 months, and 18.1 months for patient with stage I, II, and III disease, respectively. Patients who received any adjuvant treatment (chemotherapy and/or radiation, and/or hormonal treatment) had a trend (that was not statistically significant) toward longer mDFS than those without adjuvant treatment (29.7 vs. 14.1 months, *p* = 0.26). (Table 2)

**TABLE 2:** Disease‐free survival according to adjuvant treatment and FIGO stage

Treatment	mDFS	*p* value
Adjuvant treatment versus No treatment	29.7 versus 14.1	0.26
Adjuvant chemotherapy versus No treatment	29.7 versus 14.1	0.20
Stage I (*n* = 43) Adjuvant chemotherapy versus No treatment	29.7 versus 16.7	0.68

**TABLE 3:** Baseline characteristics according to adjuvant chemotherapy

	Adjuvant chemotherapy(*n* = 40)	No adjuvant chemotherapy(*n* = 28)
FIGO stage, *n* (%)		
I	23 (23 of 43, 53.4%)	20 (20 of 28, 71.4%)
II	10 (10 of 40, 25%)	2 (2 of 28, 7.1%)
III	5 (5 of 40, 12.5%)	1(1 of 28, 3.5%)
Missing FIGO staging	2 (2 of 40, 5%)	5 (5 of 28, 17.8%)
Median tumor size, cm (range)	10.5 (4–21)	10.5 (3–24.5)
Missing data	4	7
Median mitotic rate, per10HPF (range)	19 (1–60)	10 (1–63), *p* value^a^ = 0.26
Missing data	9	7
Morcellated specimen, *n*	7 (7 of 40, 19%)	4 (4 of 28, 19%)
Margin, *n* (%)		
R0	20 (20 of 40, 50%)	18 (18 of 28, 64%)
R1	7 (7 of 40, 17.5%)	0 (0)
R2	3 (3 of 40, 7.5%)	1 (1 of 28, 3.5%)
Cannot assess margin status	10 (10 of 40, 25%)	9 (9 of 28, 32%)

a No statistical difference in a median value between two groups.

**TABLE 4:** Cox proportional hazard models for disease‐free survival

Independent factors	Univariable model		Multivariable model	
	HR (95% CI)	*p* value	HR (95% CI)	*p* value
Tumor size >10 cm versus ≤10 cm	2.17 (1.12–4.22)	0.021	1.91 (0.93–3.94)	0.07
Mitotic rate >10/mm^2^ versus ≤10/mm^2^	2.47 (1.16–5.27)	0.019	1.86 (0.64–5.40)	0.25
Grade 3 versus Grade 1–2	2.62 (1.07–6.43)	0.035	1.50 (0.43–5.15)	0.51

**FIGURE 1:**
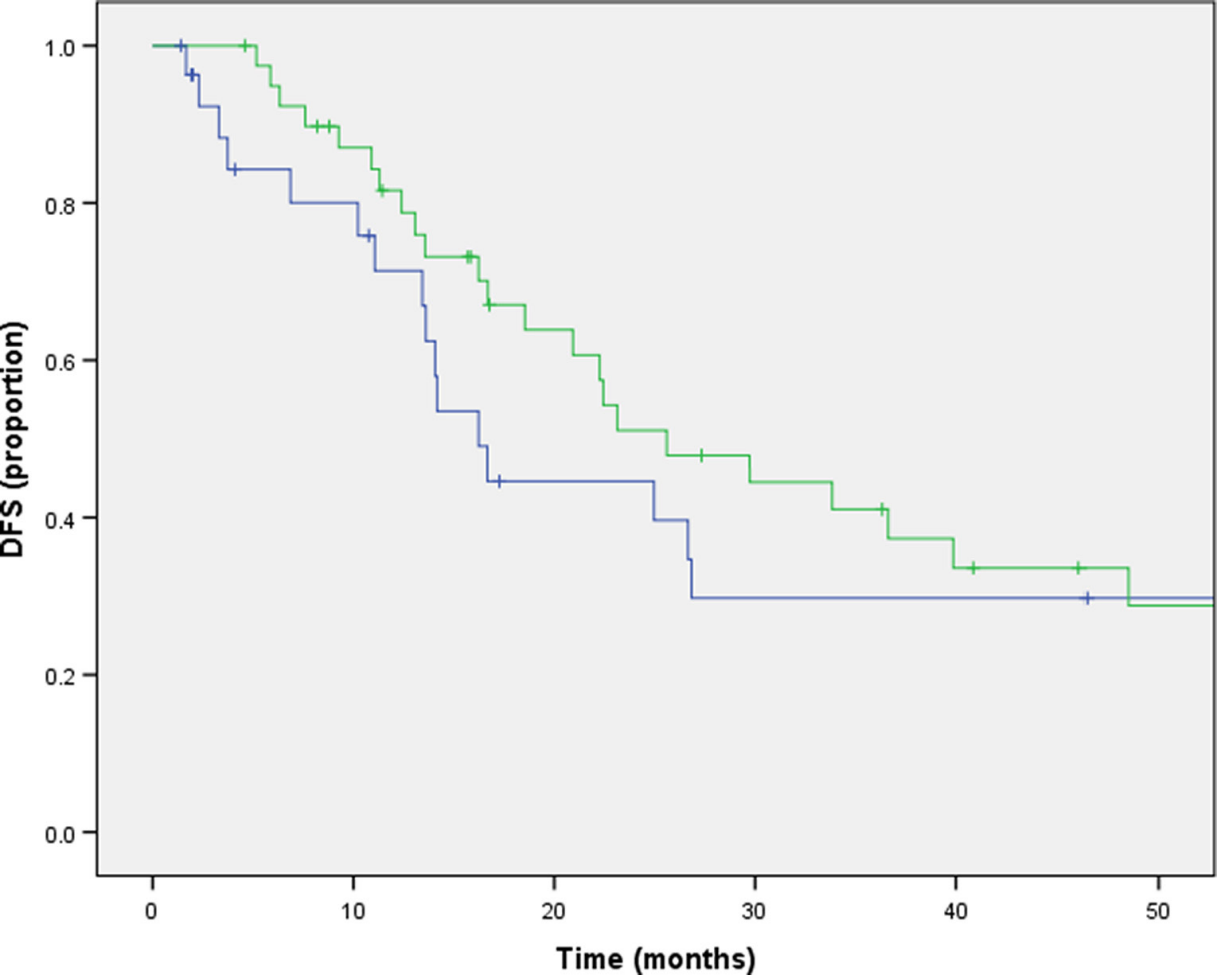
Kaplan–Meier curves for disease‐free survival according to adjuvant chemotherapy (green and blue line = adjuvant and no adjuvant chemotherapy, respectively)

Patients who only received adjuvant chemotherapy had a longer, but not statistically significant mDFS compared to those who did not receive any adjuvant treatment (22.2 vs. 14.1 months, *p* = 0.18). (Table 2) (Figure 1) The 2‐year DFS rate in patients who received any adjuvant treatment was 51%, and 29% in patients without any adjuvant treatment (*p* log rank = 0.051).

Eight of 25 (32%) patients in the non‐adjuvant arm had recurrences within 6 months compared to only 3 of 43 (6.9%) patients in the adjuvant group. Subgroup analyses of patients with stage I disease showed that, though there was a trend toward higher mDFS in the chemotherapy group, it was not statistically significant (29.7 vs. 16.6 months, *p* = 0.68). Univariate analyses found that the independent prognostic factors for worse DFS included tumor size larger than 10 cm, a mitotic rate over 10/10HPF, and tumor grade 3 (vs.grade1‐2) (Table 4). These factors did not significantly correlate with prognosis in multivariate analysis. The missing numbers of patients in equation for multivariate analysis was 33%.

In the 45 patients who relapsed, subsequent treatments included surgical resection for limited recurrent disease in 17 patients, and combined resection with systemic therapy in 11 patients. Twelve of 28 (42%) patients in the non‐adjuvant chemotherapy group received subsequent gemcitabine/docetaxel and/or doxorubicin after disease relapse. Twenty‐eight patients with widespread disease not amendable to curative surgery received palliative systemic therapy. There was no significant difference in time to second recurrence in the 17 patients whom underwent surgery for loco‐regional disease with subsequent adjuvant chemotherapy compared to those with no adjuvant chemotherapy.

## 4 DISCUSSION

In this study, we reported on the outcomes of 68 uLMS, FIGO stage I‐III patients who were seen at UCLA from 2000 to 2020. Most of the patients were FIGO stage I, and underwent TAH‐BSO. The patient and tumor characteristics including age, median tumor size, and median mitotic rate were comparable with historical series.^3^, ^19^, ^20^ In total 14.6% of tumors were French Federation of Cancer Centers Sarcoma Group (FNCLCC) grade I and II. However, according to the Stanford criteria, LMS should be regarded as intrinsically high grade.^21^ In this study, low‐ and intermediate‐grade leiomyosarcoma were reported based on grading system, but it did not appear to affect prognosis nor survival. Baseline characteristics and tumor factors such as mitotic rate, size, and age were reported as independent prognostic factors for survival.^10^ We found that tumor size, grade, and mitotic rate were found to be potential prognostic factors for shorter DFS in univariate, however, none of these factors were statistically significant in multivariate analysis in our study. This possibly could be due to the small sample size and to the 33% of total missing data.

In our study, the recurrence rate was 66%, which is within the range 45%–82% reported in the literature.^3^, ^19^, ^22^ Adjuvant treatment in operable uterine sarcoma remains controversial and there is no level 1 evidence to support its use. The rarity of this disease has made it difficult to accrue into larger randomized studies. However, single‐arm studies by Hensley et al., which included stage I‐II uLMS patients, demonstrated 2‐year DFS rates as high as 78% among those whom received four cycles of adjuvant gemcitabine and docetaxel followed by four cycles of doxorubicin, and 59% in stage I‐II whom received four cycles of adjuvant gemcitabine and docetaxel.^13^, ^16^ These studies suggests that there may be potential for benefit of adjuvant chemotherapy in resected uLMS, however, they were not designed to show superiority to no therapy.

Moreover, Pautier et al. demonstrated a significantly higher 3‐year DFS and OS in patients receiving adjuvant chemotherapy plus radiation compared to the radiation‐only.^23^ Of note, this study included all types of uterine sarcomas, and did not report on the survival outcome of the uterine leiomyosarcoma subgroup.

On the contrary, Littell et al. reported a lack of survival advantage in patients with stage I disease who received gemcitabine and docetaxel.^12^ Our study also showed no significant DFS difference between the adjuvant versus non‐adjuvant chemotherapy subgroups of stage I patients.^12^ However, the higher number of stage II and III patients in the chemotherapy arm could have skewed the DFS outcome in our study.

To address the critical question about the benefit of adjuvant chemotherapy in uLMS, the Gynecology Oncology Group ran the GOG‐0277 trial: a two‐arm, open‐label, randomized phase III superiority trial of gemcitabine plus docetaxel followed by doxorubicin versus observation in women with uterus‐limited, high‐grade LMS.^16^ Unfortunately, this trial closed early due to accrual futility. The descriptive data of this study showed differences in the estimated mean recurrence‐free survival between the two groups was 3.4 months, but the sample size was quite small. Our study demonstrated a 16‐month difference between adjuvant versus non‐adjuvant groups in stage I patients; however, this was not statistically significant.

According to meta‐analysis conducted by Rizzo et al. including uLMS, adjuvant chemotherapy did not reduce loco‐regional recurrence, distant recurrence and overall recurrence in early stage uLMS. Moreover, none of subgroups seemed to have benefit from adjuvant chemotherapy.^24^

This study has five main limitations. The first, is its small number of patients. Second, the non‐standardized adjuvant chemotherapy regimens as well as not having a uniform number of cycles. Third, as aforementioned, the significantly higher number of stage II and III patients in the adjuvant therapy group could have skewed the DFS by selection bias resulting in shorter than expected DFS in the chemotherapy arm. Fourth, 33% of missing data for multivariate analysis caused statistical problem to determine prognostic factors for shorter DFS. Finally, we could not evaluate the overall survival due to incomplete follow‐up data outside our hospital. However, the overall survival which was confounded by various subsequent approaches may not be an appropriate endpoint. Moreover, since this study did not reveal statistically significant DFS with adjuvant therapy, it would be very unlikely that this study population would have any overall survival benefit.

The strengths of our study included the institutional preference of six cycles of gemcitabine and docetaxel made the data on survival result quite homogeneous. Since uLMS is a rare sarcoma, the total of 68 patients can be an appropriate number to guide the oncology practice.

## 5 CONCLUSION

The non‐adjuvant group had a higher rate of disease relapse within 6 months, however, DFS differences between the adjuvant and non‐adjuvant groups was not statistically significant. With this study, we were not able to substantiate the benefit of adjuvant chemotherapy, and thus, cannot recommend it as a standard approach for all patients with resected uLMS.

## CONFLICT OF INTEREST

The authors declare that they have no conflict of interest.

## AUTHOR CONTRIBUTIONS

All authors contributed to the conception and design of this review. Literature search and selection were performed by Jomjit Chantharasamee and Karlton Wong. Data extraction and quality evaluation were performed by Jomjit Chantharasamee, Pasathorn Potivongsajarn, Amir Qorbani, Neda Motamed, and Arun Singh. The data analysis was performed by Jomjit Chantharasamee and Arun Singh. The first draft of the manuscript was written by Jomjit Chantharasamee and Karlton Wong, and all authors commented on previous versions of the manuscript. All authors read and approved the final manuscript.

## ETHICS STATEMENTS

This research was reviewed and approved by the *institutional review boards (IRB) of the University of California, Los Angeles*.

## Data Availability

The data that support the findings of this study are available from the corresponding author upon reasonable request
